# Emerging Trends in Artificial Intelligence-Integrated Biochip Technologies for Biomedical Applications

**DOI:** 10.3390/mi17050623

**Published:** 2026-05-19

**Authors:** Muniyandi Maruthupandi, Nae Yoon Lee

**Affiliations:** 1 Department of BioNano Convergence, Gachon University, 1342 Seongnam-daero, Sujeong-gu, Seongnam-si 13120, Gyeonggi-do, Republic of Korea; 2 Department of BioNano Technology, Gachon University, 1342 Seongnam-daero, Sujeong-gu, Seongnam-si 13120, Gyeonggi-do, Republic of Korea

**Keywords:** artificial intelligence, machine learning, deep learning, biochip, bacteria, virus, diabetes, diagnostics, neurological disorders, drug delivery

## Abstract

Neurological disorders, diabetes, cancer, and infectious diseases remain major global health concerns, particularly in low- and middle-income countries with insufficient access to accurate and rapid diagnostics. Conventional biochip sensing platforms, while effective, are often constrained by complex instrumentation and have limited capability for handling complex and large datasets. This review aims to address these limitations by evaluating the integration of artificial intelligence (AI) with biochip technology improve biomedical diagnostics. We systematically analyze recent advances in AI-integrated biochips, such as spectroscopic, paper-based, lab-on-chip, and microfluidic platforms integrated with reinforcement learning, machine learning, and deep learning models. These pre-trained AI models simplify pattern recognition, feature extraction, and automated data processing from a variety of biosensor outputs, such as electrochemical, fluorescence, and colorimetric signals. The reviewed studies indicate improved real-time diagnostic sensitivity and accuracy across biomedical applications. Overall, we discuss ongoing challenges and future perspectives toward explainable, robust, and smartphone-assisted AI-integrated biochips for rapid and accurate diagnostics. The review offers a comprehensive overview of AI-integrated biochips to support effective disease detection and clinical decision-making.

## 1. Introduction

### 1.1. Sensing Challenges in Biomedical Diagnostics and Biochip Technologies

Infectious diseases, cancer, diabetes, and neurological disorders remain key global health challenges worldwide. Biomedical diagnostics require rapid, accurate, and accessible diagnostic tools for effective early disease detection, prevention, and treatment monitoring. However, traditional diagnostic methods often require advanced equipment and are affected by operator-dependent variability, which limits their usefulness for point-of-care testing (POCT) applications.

**Figure 1:**
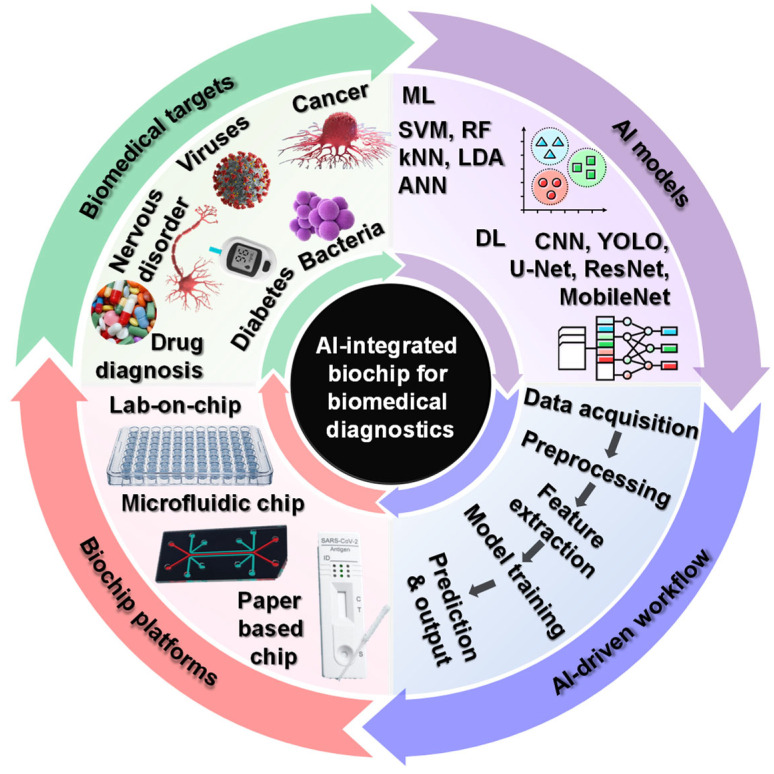
Schematic representation of the AI-integrated biochip platforms for biomedical applications.

Infectious diseases are caused by pathogenic microorganisms, including bacteria, viruses, fungi, and parasites. Bacteria present in air, water, and food are highly dangerous to human health. In particular, bacterial diseases, such as urinary tract infections (UTIs), tuberculosis, pneumonia, and cholera, cause major health concerns and mortality worldwide. The World Health Organization (WHO) reported 1652 pathogens from 28 viral families (Figure 1) [1,2,3]. Viruses also pose challenges owing to their rapid mutation and high transmissibility [4,5]. Coronaviruses, influenza viruses, filoviruses, human papillomavirus (HPV), African swine fever virus, and flaviviruses are major high-risk viruses worldwide. Severe acute respiratory syndrome coronavirus 2 causes Coronavirus disease 2019 (COVID-19). Since December 2019, COVID-19 has killed about 6.9 million people worldwide (Figure 1) [1,6,7]. Cancer is characterized by the rapid growth of abnormal cells that grow beyond their normal boundaries, invade nearby tissues, and spread to other organs. According to the WHO, cancer accounted for nearly 10 million deaths in 2020, with lung, colon, liver, stomach, and breast cancers among the most common causes. Cancer-causing infections such as HPV and hepatitis are responsible for approximately 30% of cancer cases in low- and middle-income countries (Figure 1) [8,9].

Diabetes is a serious chronic metabolic disorder that occurs when blood glucose levels increase because of insufficient insulin production. Diabetes is one of the fastest-growing global health concerns, especially type 2 diabetes. The International Diabetes Federation reported that more than one in ten adults are living with diabetes, and around 537 million adults were living with diabetes in 2021. Over time, diabetes can lead to cardiovascular disease, kidney failure, and neuropathy, making early diagnosis crucial (Figure 1) [10,11]. Neurological disorders involve abnormalities of the central and peripheral nervous systems, such as Alzheimer’s disease, multiple sclerosis, Parkinson’s disease, epilepsy, muscular dystrophy, and peripheral neuropathy. The disorders can result in progressive cognitive and motor impairment, significantly affecting quality of life. Neurological disorders are a global health problem that affects health, the economy, and social life. The WHO estimated that neurological disorders affected approximately 3.4 billion people in 2021. These disorders caused around 11.8 million deaths, 162 million years of disability, and 272 million years of life lost worldwide (Figure 1) [12,13]. The increasing global burden of infectious diseases, cancer, diabetes, and neurological disorders highlights the need for novel approaches for drug delivery, discovery, and screening. Traditional drug discovery and diagnosis methods are time-consuming, expensive, and often slow in responding to viral infections and cancers [14,15]. These factors collectively highlight the urgent need for rapid, reliable, and field-deployable POCT biochip sensing platforms for biomedical applications (Figure 1).

### 1.2. Limitations of Conventional Biochip Platforms

Conventional biochip platforms, such as lab-on-chips (LOC), microfluidic chips, paper-based chips, tissue/cell microarrays, protein microarrays, and DNA microarrays, are widely used for biomedical diagnostics, drug screening, and pathogen detection. These biochip platforms are commonly integrated with optical and electrochemical detection techniques, such as fluorescence spectroscopy, colorimetry, surface-enhanced Raman spectroscopy (SERS), fluorescence imaging, impedance spectroscopy, and voltammetry. Although these systems offer advantages such as miniaturization, low sample consumption, and rapid analysis, several limitations still restrict their practical use in clinical and POCT applications [16,17,18].

The major limitations of conventional biochip platforms are their susceptibility to non-specific binding and background interference, particularly in complex biological samples such as urine, saliva, serum, and blood. Such interferences can decrease the signal-to-noise ratio and lead to false-positive or false-negative results. Typically, protein and DNA microarrays need highly specific probe immobilization, stringency hybridization conditions, and multiple purification steps, which reduce reproducibility and increase complexity. Conventional LOC and microfluidic biochips face challenges related to sample preparation, fluid control, and device-to-device variability. Accurate analytical performance requires precise control of fluid flow and reagent mixing with minimum reaction time. However, variations in channel design, sample viscosity, evaporation, and capillary flow can cause variation in signal response. Paper-based biochips are attractive for low-cost diagnostics, but their performance may be impaired by non-uniform sample flow, poor quantitative accuracy, weak signal intensity, and environmental effects such as humidity and temperature.

Another notable limitation is the difficulty of multiplex detection and interpretation of large datasets. Although biochips can analyze multiple targets, cross-reactivity between probes, signal overlap, and complex data processing can reduce analytical reliability. Furthermore, manual image interpretation may introduce human errors and increase variability. Moreover, many conventional biochip systems require external instruments, trained personnel, and laboratory-based signal analysis, which limits their direct use in resource-limited areas and on-site diagnostics. Therefore, despite significant progress in conventional biochip technologies, their practical applications remain limited by non-specific binding, background interference, limited sensitivity, poor reproducibility, signal variability, manual handling errors, complicated multiplexing, and insufficient suitability for real-time POCT. These limitations highlight the need for an advanced biochip platform integrated with artificial intelligence (AI)-integrated signal analysis, automated image interpretation, and improved analytical sensitivity for reliable biomedical diagnostics [19,20,21].

In addition to analytical limitations, diagnostic turnaround time (TAT) and test cost remain major challenges for routine clinical decision-making. Conventional bacterial culture is relatively inexpensive, costing approximately USD 10–40 per test, but requires 24–72 h for results, while antimicrobial susceptibility testing may need an additional 24–48 h and costs around USD 20–60 [22,23]. PCR/RT-PCR provides faster results, within 1–4 h, but the cost is higher, generally around USD 50–150 per test, due to sample preparation, reagents, instruments, and trained personnel [24]. Cancer diagnosis, including histopathology, immunohistochemistry, imaging, and molecular profiling, usually requires hours to days and may cost USD 100–500 [25]. Diabetes testing is comparatively low-cost (USD 1–2), especially glucose-strip testing, whereas neurological diagnosis and therapeutic drug monitoring remain expensive (USD 30 to 1000) because they depend on centralized imaging, immunoassay, HPLC, LC-MS, and ELISA facilities [26]. Therefore, future AI-integrated biochip platforms should reduce both TAT and cost per test while minimizing instrument dependence and skilled-labor requirements for affordable POCT diagnostics [22,23,27,28] (Table 1).

### 1.3. Artificial Intelligence-Integrated Biochip Platforms

AI has emerged as an effective analytical tool for addressing the limitations of conventional optical and electrochemical biochip sensors. While the early theoretical basis of AI emerged from Alan Turing’s foundational concepts in the 1950s, recent developments have enabled its practical use in biosensor applications. In this context, AI-integrated biochips can learn directly from raw datasets, undergo training, recognize signal patterns, and provide more consistent interpretation of signals, such as viscosity, color, image patterns, fluorescence, and electrochemical responses. However, AI-integrated biochips are typically pre-trained and without prior training. Incorporating deep learning (DL), reinforcement learning (RL), and machine learning (ML) models can support rapid and reliable analysis, which is valuable for applications such as infectious disease diagnosis, cancer screening, diabetes monitoring, neurological disorders, and drug delivery and diagnosis [29,30,31]. Based on learning models, AI algorithms used in biochip systems can be generally classified into supervised, unsupervised, semi-supervised, and RL approaches. Among ML-based supervised learning algorithms, support vector machines (SVM), random forest (RF), linear discriminant analysis (LDA), k-nearest neighbors (kNN), and artificial neural networks (ANN) are extensively employed for classification and regression tasks (Figure 1 and Table 2). These models depend on handcrafted features derived from optical, colorimetric, fluorescence, and electrochemical signals and require well-labeled datasets. For example, SVM constructs optimal hyperplanes for class separation, while RF improves prediction robustness through ensemble decision trees. Similarly, kNN classifies samples based on similarity metrics, and LDA enhances class separability through linear projections. Overall, these models are effective for structured dataset, their performance depends heavily on feature engineering and parameter optimization, and they may struggle with highly complex data (Table 2) [3,32,33].

In comparison, DL-based supervised learning algorithms directly learn hierarchical representations from raw data inputs. Convolutional neural networks (CNNs) are used for extracting spatial features from color and imaging datasets, while You Only Look Once (YOLO) allows real-time object detection. U-shaped network (U-Net) architectures are highly effective for pixel-level segmentation of reactive regions, while residual networks facilitate deep feature learning through residual connections. The MobileNet model offers an on-site, lightweight alternative suitable for portable and smartphone-integrated biochip systems. The DL models require large and complex datasets and significant computational resources, such as graphics processing unit (GPU)-based acceleration, but they provide superior performance in handling complex and high-dimensional biosensor data (Figure 1) [3,34,35]. Beyond DL and ML, RL algorithms such as Q-learning and deep Q-networks (DQN) are emerging as promising tools for adaptive biosensor platforms. RL enables biochips to optimize sensing protocols through reward-based learning, making them suitable for dynamic and real-time decision-making. However, RL models require well-defined reward mechanisms and involve high computational complexity (Table 2) [36,37]. Overall, the selection of suitable AI models in biochip applications depends on data type, data size, computational resources, and application requirements. Data preprocessing is an essential step before AI-based biosensing analysis because raw biochip signals often contain noise, background variation, matrix interference, and device-to-device variability. Therefore, preprocessing methods such as denoising, baseline corrections, background subtraction, normalization, region-of-interest selection, and feature engineering are important for improving signal quality. These steps help extract reliable features from optical, colorimetric, fluorescence, spectroscopic, and electrochemical biochip data, thereby improving accuracy, reproducibility, and generalization. ML algorithms are well-suited for structured datasets with limited samples, whereas DL algorithms are effective in processing large, complex, and unstructured data. RL provides an additional layer of adaptability for intelligent and autonomous sensing systems. Therefore, the AI-integrated biochips represent a transformative step toward next-generation smart diagnostics with enhanced sensitivity, accuracy, real-time analysis, and on-site monitoring (Table 2) [38].

### 1.4. Scope of This Review

Despite rapid developments in conventional and AI-integrated biochip platforms for biomedical analysis, a critical and integrative assessment that systematically examines the limitations of conventional biochip platforms and the potential AI-driven solutions remains limited. In this review, we provide a comprehensive and challenge-focused analysis of conventional biochip systems, primarily focusing on limitations related to selectivity, signal variability, data interpretation, and real-time applicability. Furthermore, we discuss how ML and DL enable automated feature extraction, enhanced pattern recognition, and improved analytical reliability. The scope of this review covers AI-integrated biochip platforms for biomedical applications, with a focus on infectious diseases, cancer, diabetes, neurological disorders, and drug delivery and diagnostics, which remain underexplored in an exciting review (Figure 1). Recent developments reported over the past six years are systematically summarized, highlighting the integration of AI with fluorescence spectroscopy, colorimetry, surface-enhanced SERS, fluorescence imaging, impedance, and voltammetry-based biochip platforms. In addition, this review presents an end-to-end workflow and AI-integrated biochip platforms encompassing data acquisition, preprocessing, feature extraction, model training, and decision-making processes. Finally, limitations and future perspectives are outlined to guide the development of reliable, high-performance, and POCT biochip platforms for next-generation biomedical diagnostics.

## 2. AI-Integrated Biochips in Biomedical Applications

In recent years, the combination of biochip platforms and AI has gained significant momentum for the use of biochips in biomedical applications. Modern biochip platforms are facilitated by advancements in microfabrication and device miniaturization by integrating sample handling, rapid detection, signal processing, and wireless communication into compact systems. Owing to these advances, modern biochips are used for POCT and high-throughput dataset analysis [39,40]. However, the larger and more complex datasets produced by biochip platforms often present challenges in data interpretation and results analysis in clinical diagnostics in resource-limited areas. AI-integrated biochip platforms enables automated data analysis, pattern recognition, anomaly detection, and outcome prediction. However, AI models do not autonomously adapt and are typically pre-trained. Consequently, AI-integrated biochips platforms facilitate more rapid and reliable identification of disease diagnosis and therapeutic monitoring, including cancer diagnosis, pathogen detection, diabetes management, neurological disorders, and drug delivery [41,42]. This section provides an overview of AI algorithms that enhance the performance of biochips for diseases, therapeutic diagnoses, and monitoring.

### 2.1. AI-Integrated Biochips for Disease Diagnosis

AI-integrated biochips are emerging as a promising platform for disease diagnosis. Their main advantages include improved data processing, image analysis, automated results interpretation, sensor optimization, and workflow standardization with typically pre-trained models. This section discusses how AI-integrated biochips have been applied to wide disease diagnostics, such as cancer, diabetes, and pathogen detection. Reported studies have demonstrated their use in liquid biopsy analysis, exome profiling, pathology imaging, and disease subtype classification using microfluidic, SERS, fluorescence spectroscopy, colorimetric, electrochemical sensing, and fluorescence imaging platforms. This section is organized into three major application areas, namely, pathogen detection, cancer diagnosis, and diabetes monitoring.

#### 2.1.1. Pathogen Detection and Infectious Disease Screening

**Figure 2:**
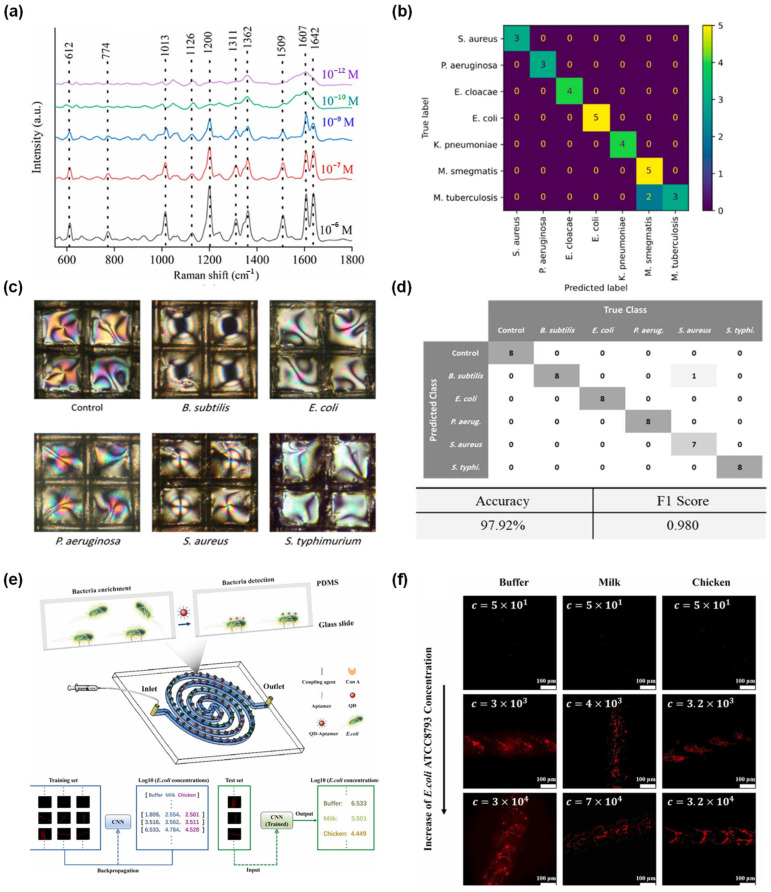
(**a**) SERS chip sensitivity by considering SERS spectra of R6G for micro- to pico-molar concentrations, reprinted with permission from [44], copyright 2023 American Chemical Society (ACS). (**b**) SNN for prediction and classification of bacteria based on SERS data, reprinted with permission from [44], copyright 2023 ACS. (**c**) Photographs of bacterial strain optical patterns of the various bacterial strains in milk, reprinted with permission from [47], copyright 2024 ACS. (**d**) Bacterial strain optical pattern classification using SVM analysis, reprinted with permission from [47], copyright 2024 ACS. (**e**) Schematic microfluidic biochips and CNN-based model used for rapid detection of *E. coli*, reprinted with permission from [48], copyright 2025 Elsevier. (**f**) Photographs of fluorescence markers for *E. coli* across different food categories at varying concentrations, reprinted with permission from [48], copyright 2025 Elsevier.

Rapid and accurate detection of pathogens and infectious disease screening is critical for containing outbreaks of infectious diseases and preventing their spread. AI-integrated biochips are relevant for pathogen detection because they can address challenges such as biological complexity and complex signal interpretation. ML and DL models are used to classify viscosity, flow, images, color, spectral, volatile metabolite patterns, and DNA signals, often converting complex signals into practical diagnostic outputs. A smartphone camera was used to record the flow velocity, and an SVM classifier converted flow fingerprints into species-level classifications from images. This approach was demonstrated by Kim et al. (2021) [43], who introduced a smartphone-based paper microfluidic platform inspired by human sensory recognition rather than conventional lock-and-key baroreceptors. The platform used peptide-conjugated polystyrene particles loaded onto paper microfluidic chips. The interaction between the peptide and bacteria altered particle aggregation, which subsequently changed the capillary flow behavior through the paper-based device. Smartphone-based SVM could continuously perform non-contact monitoring for *Escherichia coli* (*E. coli*), *Staphylococcus aureus* (*S. aureus*), *Salmonella typhimurium* (*S. typhimurium*), *Enterococcus faecium* (*E. faecium*), and *Pseudomonas aeruginosa* (*P. aeruginosa*) in field water samples. The SVM model reached 93.3% accuracy using flow monitoring within less than 6 s and a response time of less than 10 min [43]. However, the mechanism of peptide-bacteria interaction was not fully resolved, and performance depended strongly on the paper substrate, indicating that material choice remains a practical limitation. Similarly, Das et al. (2023) [44] developed a SERS nanowire chips for pathogen detection as a label-free spectroscopic platform and focused on both species-level and strain-level bacterial classification with antimicrobial resistance (AMR)-related strains. The biochips consisted of silver (Ag)-coated nanowires fabricated by metal-assisted chemical etching, resulting in a large-area plamonic substrate for SERS. A Siamese neural network (SNN) was used as a deep feed-forward network with several layers to analyze limited sample numbers and complex datasets. The platform classified 12 bacterial species, including *S. aureus*, methicillin-resistant *S. aureus*, *E. coli O157:H7*, and *E. coli ER2738*, linked to tuberculosis and UTIs. This system robustly and reproducibly detects and classifies bacterial species through SERS changes. Specifically, the system achieved a limit of detection (LOD) of 100 colony-forming units (CFU)/mL, enabling real-time detection in urine samples. Overall, these studies demonstrate the effectiveness of SNN in extracting SERS datasets with low LOD, label-free sensing, and AMR detection (Figure 2a,b) (Table 3) [44].

Wang et al. (2024) [45] implemented a metabolism-triggered sensor array study that addressed a common weakness of chemical-nose systems: their dependence on non-specific interactions that are easily disturbed by environmental conditions. To overcome this limitation, the authors built a new sensing strategy with a lab-built fluorescence spectrometer. Pathogens were metabolically labeled with 3-azido-D-alanine at different pH conditions and time intervals, and reacted with clickable upconversion nanoparticles (UCNPs) to produce four-dimensional fluorescence outputs. Integration of the PCA model with the fluorescence platform enabled highly selective detection of *S. aureus*, methicillin-resistant *S. aureus*, *E. coli O157:H7*, and *E. coli ER2738* with 100% accuracy and a response time of 120 min. The interesting feature is that this platform can identify unknown species outside the training set and assign them to a broader phenotype class. This is a strong conceptual advance because it provides better robustness for complex and larger pattern recognition systems. The main limitation of this platform, as implied by the study and training design, is that the demonstrated panel was controlled and focused on model bacteria, and required a relatively long response time, so broader real-world validation across more complex pathogens would be needed [45]. Jia et al. (2024) [46] developed a deep feed-forward neural network DFFNN-enabled paper chromogenic array for bacterial diagnosis through volatile organic compounds (VOCs) without direct contact with the foodborne pathogen The platform used a 3 × 3 paper chromogenic array with dyes that responded to a specific VOC mixture, and the DFFNN model analyzed the time-dependent red–green–blue (RGB) changes. The sensing system used LFA strips for detecting *L. monocytogenes*, *Salmonella*, and *E. coli O157:H7*, including individual and multiplex contamination scenarios in chicken samples. The DFFNN model detected inoculum levels as low as approximately 1 log CFU/g, achieving approximately 90% accuracy with about 80% accuracy in the chicken sample. The strengths of this platform are portability, low cost, and non-contact operation. The main limitations are its strong dependence on temperature and VOC accumulation time (Table 3) [46].

The liquid crystal optical sensor array enhanced with cysteine-functionalized Ag nanoparticles (AgNPs) for pathogen detection in food and water was reported by Mousavizadegan et al. (2024) [47]. The biochip relied on LC texture changes under polarized light, while the cysteine-functionalized AgNPs enhanced bacteria–sensor interactions and optical contrast. Imaging of the resulting patterns was performed using an SVM model to classify five bacteria, such as *Bacillus cereus (B. cereus*), *E. coli*, *P. aeruginosa*, *S. aureus*, and *S. typhimurium*. The SVM model enabled the detection of about 10 log CFU/mL, achieving an accuracy of 98.89% within a linear range from 10 to 10^6^ CFU/mL in the milk, water, and juice samples. The SVM model performance in water and juice remained high, while milk caused more errors, likely because of the high protein and lipid background (Figure 2c,d) [47]. Ma et al. (2025) [48] reported DL-enhanced staggered herringbone double-spiral microfluidic biosensor-targeted ultrasensitive detection of foodborne *E. coli*. The biochip integrated encirhment, capture, detection, and release in a staggered herringbone double-spiral microfluidic design. A quantum-dot aptamer probe provided fluorescence images with varying intensities, based on the *E. coli* concentration. A ResNet-18-based CNN directly estimated bacterial concentration from fluorescence images, reducing reliance on conventional image analysis. The CNN-integrated biosensor showed a linear range from 10 to 3 × 10^6^ CFU/mL, capture efficiency up to 100% at low concentrations, and a LOD of 2 CFU/mL within 90 min. The novelty of the CNN-integrated biosensor lies in combining a highly engineered enrichment chip with image-based DL that actively filters background and low-quality signals; multiplex pathogen detection was not demonstrated (Figure 2e,f) [48]. Tran et al. (2025) [49] reported a rapid phenotypic drug susceptibility testing platform for the *Mycobacterium tuberculosis* variant *M. bovis* BcG using microfluidic single-cell imaging and deep neural network-based image segmentation. The method monitored mycobacterial growth inside microchambers and used omnipose-based segmentation models to quantify cell growth under antibiotic exposure. The platform enabled rapid susceptibility for slow-growing mycobacteria within a much shorter time than conventional culture-based TB drug susceptibility testing. The main strength of this work is the combination of microfluidic, time-lapse microscopy, and DNN-assisted analysis for faster TB-related drug resistance diagnosis. However, it was mainly validated using *M. bovis* BCG and *M. smegmatis* model systems, and high-density cell clumps remained difficult for accurate signal-cell segmentation [49]. Marino et al. (2025) [50] introduced a culture-free microfluidic and DL-based method for detecting bacteria directly from blood samples, aiming at rapid sepsis diagnosis. The system combined bacterial enrichment, microfluidic trapping, time-lapse phase-contrast imaging, and DL models such as ResNet-18 and DinoV2 for automated bacterial detection. Among these, DinoV2 showed the best performance, achieving an F1 score of 93.1% at 70 min, and ResNet-18 detected an LOD of 1 to 10 CFU/mL within 2 h, while negative sterile-blood controls showed zero bacterial detections. However, poor isolation of *S. aureus* from blood was identified as a major limitation, which may lead to false-negative results in some sepsis cases [50]. Agnihotri et al. (2025) [51] developed a droplet microfluidic platform for detecting rare antibiotic-resistant subpopulations in *E. coli*, particularly for heteroresistance analysis in bloodstream infection-related isolates. The system encapsulated bacterial populations into droplets and detected resistant subpopulation growth based on antibiotic-induced droplet shrinkage, which was quantified by microscopy. The method could detect resistant subpopulations at frequencies as low as 10^−6^ and required far fewer droplets than conventional single-cell droplet AST approaches. However, this paper is mainly a microfluidic phenotypic diagnostic platform rather than an AI-integrated biochip. In addition, the method requires longer incubation and a specialized droplet microfluidic setup, which may limit its immediate POCT application [51]. The hand-driven microfluidic clustered regularly interspaced short palindromic repeats (CRISPR) chip developed by Xu et al. (2025) [52] enabled viral nucleic acid screening and focused on high-risk HPV-16 and HPV-18 in clinical samples. The chip was fabricated by combining a multilayer polymethyl methacrylate (PMMA) microfluidic chip with sequential reagent release and three parallel working units, and a smartphone microimaging system combined with the R-CHIP ResNet-18 model for automated interpretation. The AI-integrated biochips achieved sample preprocessing-to-result analysis in about 60 min and achieved an LOD of 10^−18^ M for HPV-16 and HPV-18. The validation with multiplex polymerase chain reaction (PCR) reached 98 to 99% accuracy for detecting HPV-16 and HPV-18 across 300 tests on 100 cervical swab samples, while the smartphone AI model achieved 94% overall accuracy for four-class image categorization. The biochip platform is novel because it combines manual portability, molecular amplification, CRISPR specificity, and AI-assisted readout in one community-screening workflow (Table 3) [52].

Collectively, these reported studies suggest that AI-integrated biochips for pathogen detection can be classified into three main groups, namely paper-based, spectroscopic, and integrated molecular chips. Paper-based and smartphone-assisted biochips are attractive for low-cost and field-deployable testing because they analyze visual changes in flow, color, or texture. However, their performance can be affected by substrate variability, lighting conditions, image quality, and reproducibility issues. In contrast, SERS, fluorescence, and spectroscopic biochips generally provide higher sensitivity and lower LOD, but they require optical instrumentation, controlled signal acquisition, and careful spectral preprocessing. Integrated molecular chips, such as the R-CHIP platform reported by Xu et al. (2025) [52] for HPV detection, show strong diagnostic potential due to their integrated workflow design, rapid analysis, clinical relevance, strong agreement with PCR results, smartphone-assisted readout, and suitability for resource-limited areas. However, challenges remain in chip fabrication, reagent stability, multiplex validation, and real-sample testing. Importantly, many studies report high AI accuracy using limited or controlled datasets, while external validation with diverse clinical and environmental samples is still insufficient. Therefore, future pathogen detection biochip studies should more consistently report analytical sensitivity, LOD, response time, sample type, diagnostic cost, dataset size, model validation strategy, and clinical applicability. This will be essential for improving reproducibility, reducing dataset bias, and translating AI-integrated biochips into practical POCT diagnostics.

#### 2.1.2. Cancer Diagnosis

**Figure 3:**
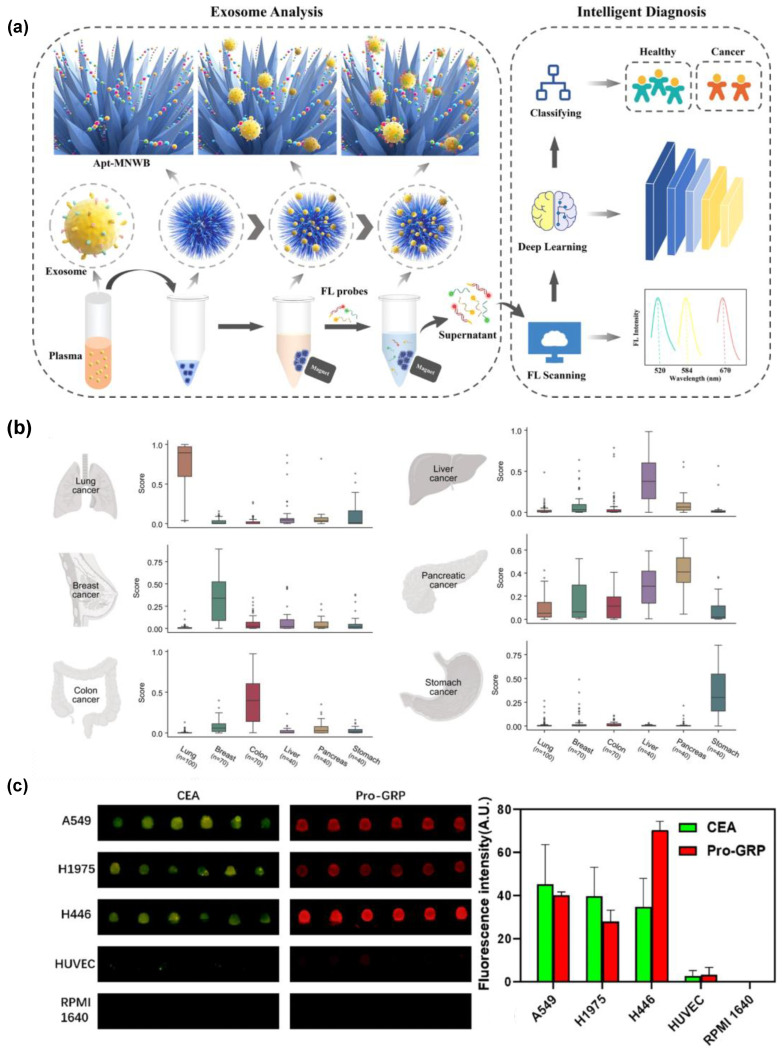
(**a**) Magnetic nanowaxberry-based simultaneous detection of exosomes and exosome proteins for the AI diagnosis of cancer, reprinted with permission from [53], copyright 2021 ACS. (**b**) AI-integrated SERS platform for simultaneous multi-cancer diagnosis through tissue of origin determination for lung, breast, colon, liver, pancreatic, and stomach cancers, reprinted with permission from [55], copyright CC BY 4.0. (**c**) Fluorescence imaging of exosomes from four cell lines for dual-tumor marker detection with YOLOv8-based automated histogram analysis of exosome tumor level, reprinted with permission from [58], copyright 2025 Elsevier.

Cancer continues to be a major global health concern, and early diagnosis plays a significant role in ensuring effective treatment. AI-integrated biochips are becoming increasingly important in cancer early diagnosis because they combine miniaturized analytical platforms with computational models. AI-integrated biochips can manage complex biological signals more consistently than manual interpretation alone. ML and DL models are used to classify cancer diagnosis, but also as a tool for chip optimization, signal interpretation, image recognition, and standardization of diagnostic readout. Recent studies support the growing use of this method in optical techniques, such as fluorescence and SERS. Ding et al. (2021) [53] developed fluorescent sensors using magnetic nanowaxberry, which uses decision tree (DT), RF, and SVM algorithms to analyze exosome concentration and exosome proteins at the same time. This platform aimed to improve cancer diagnosis by measuring both the number of vesicles and their protein content in a single assay, rather than relying on a single marker. The platform used aptamer-grafted magnetic nanowaxberry particles, which have a zinc oxide nanowire-rich surface, to capture exosomes. It then used three-color probes to quantify epidermal growth factor receptor (EGFR), epithelial cell adhesion molecule (EpCAM), and lipid-based exosomes from lung cancer. DL models were employed to extract diagnostic patterns from the assay output datasets, resulting in a 96% accuracy rate. The AI-integrated biochips design facilitated interference-resistant, multiplexed detection with elevated higher sensitivity while eliminating the necessity for individual exosome isolation. Nevertheless, the limited sample size constrains the DL models robustness and generalizability, necessitating additional validation (Figure 3a) [53]. Similarly, Ding et al. (2023) [54] developed an irregular serpentine microfluidic chip combined with magnetic nanowaxberry particles modified with a CD63 aptamer for efficient recovery of circulating exosomes from plasma. The purpose of this platform was to solve a persistent bottleneck in cancer diagnosis. AI-integrated microfluidics used for plasma exosomes were profiled using five biomarkers, including exosome concentration, EGFR, EpCAM, serum amyloid A-1 protein (SAA1), and coagulation factor V (FV), and the resulting diagnostic area under the curve (AUC) reached 0.791 for lung cancer. The novelty of the platform was in practical separation performance, with around 24-fold higher recovery with excellent purity from chip-based extraction compared to centrifugation and magnetic nanowaxberry particles to combine affinity capture with a size-exclusion effect [54]. In 2023, Shin et al. [55] reported an exosome–SERS–AI biochips study for early-stage multi-cancer detection. The AI-integrated biochips achieved one test for simultaneous detection of six types of cancers, such as lung, breast, colon, liver, pancreas, and stomach cancers, using plasma exosomes. Firstly, exosomes were isolated by size exclusion chromatography, and then analyzed on a gold nanoparticle (AuNP)-aggregated array chip that generated label-free SERS spectra. The AI component involved CNN, SVM, and a multi-layer perceptron (MLP), including a first-stage cancer presence classifier and a second-stage tissue of origin prediction framework. The system achieved an AUC of 0.970 for cancer detection in an independent test set of 520 samples for tissue of origin classification in 278 early-stage cancer patients. The final integrated model yielded 90.2% sensitivity at 94.4% specificity and predicted origin for 72% of positive patients. A key strength of this platform is its biomarker-independent evaluation of the full plasma exosome spectrum, allowing broad prescreening with possible translational value. Nevertheless, the approaches offer limited biological interpretability, and tissue-of-origin assignment was not achieved for all positive cases (Figure 3b) (Table 3) [55].

Selcuk et al. (2024) [56] reported a CNN-integrated automated immunohistochemically (IHC) human epidermal growth factor receptor 2 (HER2)-stained slide chip for diagnosing breast cancer. This platform used digitized HER2-stained breast cancer tissue images, specifically tissue microarray cover images. These images were then analyzed by a CNN model that used pyramid sampling to preserve information across different spatial scales. This platform addressed a common problem in earlier HER2 systems. It avoided the overly selective analysis of patches by capturing both cellular- and tissue-level context. The system achieved an 84.7% accuracy for breast cancer for 523 core sample images with a response time of 15 s. Although AI-integrated biochips show a pyramid-sampling approach that enhances clinically realistic analysis of heterogeneous tissues without requiring extensive pathology processing, their performance is imperfect and is limited to tissue microarray-based evaluation rather than full real-world clinical deployment [56]. In another innovative study, Nie et al. (2025) [57] developed a microfluidic chip that performed on-chip enzymatic deglycosylation of extracellular vesicles (EVs)-associated programmed death-ligand 1 (PD-L1), thereby increasing epitope accessibility before fluorescence-based detection of lung cancer. The RF- and MLP-integrated microfluidic chip was to improve the detection of PD-L1-positive EVs for non-invasive lung cancer diagnostics and immunotherapy stratification, because glycosylation can mask PD-L1 epitopes and weaken standard antibody-based arrays. Plasma samples were collected from 30 lung cancer patients and 15 healthy donors, and the biomarker was PD-L1 on EVs. The platform efficacy was good by employing a hybrid ML algorithm, namely the reTesla Stacking Ensemble (rTSE). The algorithm combined RF and MLP models. This approach was used to optimize chip geometry and flow-related mixing parameters, using data from computational fluid dynamics simulations. As a result, the optimization process enchanced the efficiency of deglycosylation and also reduced the LOD to 10^3^ EVs. This represents a significant improvement when contrasted with a conventional microfluidic approach, which achieved an LOD of 10^5^, and a microcentrifuge-based method, which demonstrated an LOD of 10^6^. The platform completed analysis in a response time of 30 min and achieved 96.7% sensitivity with 100% specificity in clinical plasma samples. The platform detected PD-L1 EVs in many tissue PD-L1-negative cases better than ELISA. The innovation stems from using AI for classification and optimizing microfluidics. However, the small size of the initial clinical group limits its immediate applicability, indicating that larger studies are needed (Table 3) [57].

Lu and colleagues (2025) [58] created a microfluidic system that allowed for the complete isolation of exosomes on a single chip, along with automated image analysis. This system combined the isolation of exosomes using immunobeads within a microfluidic chip with quantum-dot labeling of tumor markers. Afterwards, the bead–exosome units were dispersed into a microcolumn to enable optical sensing. The YOLOv8 model was used to locate individual bead units in bright-field images and transfer the coordinates for fluorescence intensity extraction. The platform examined exosomes from several cell lines, including A549, H1975, H446, and HUVEC, using CEA and Pro-GRP as tumor markers. The YOLOv8 model showed an LOD of 8.65/µL. Differences in marker levels were also observed across the cell lines, showing a strong agreement with existing marker assays. The results indicated that using a YOLOv8-integrated microfluidic device helped with the on-chip isolation, labelling, migration, and quantification of the samples. Furthermore, the use of AI reduced errors in manual reading and enhanced the detection of weak signals (Figure 3c) [58]. Moreover, Chen et al. (2025) [59] present a microfluidic-SERS study to subtype cancer, particularly non-small-cell lung cancer, focusing on a specific clinical issue using exosome fingerprints. The method used polystyrene microspheres coated with gold nanocubes and antibodies, followed by an optical SERS readout. DL, specifically the ResNet architecture, was used to classify exosome spectra that did not have labels. These spectra came from a normal lung epithelial cell line and three different non-small-cell lung cancer (NSCLC) cell lines. The system demonstrated a peak trapping efficacy of 85%, along with an overall classification accuracy of 97.88% and an AUC value of 0.95 for each category. ResNet effectively combines label-free SERS with interpretable DL methods, which helps to accurately and understandably identify subtypes. Nevertheless, its application is constrained, given that validation was performed only using exosomes isolated from cell lines, without clinical sample validation (Table 3) [59].

Overall, AI-integrated biochips for cancer diagnosis mainly rely on exosome profiling, SERS, fingerprints, fluorescence imaging, and microfluidic biomarker detection. These platforms show strong potential for improving sensitivity, enabling multiplex cancer classification, and supporting minimally invasive diagnosis. For example, blood plasma was used in the multi-cancer SERS-AI system and the PD-L1 extracellular vesicle chip, whereas some other studies used cell-line exosomes mainly for proof-of-concept validation. A biomarker-specific platform targeting EGFR, EpCAM, CEA, Pro-GRP, and PD-L1 provides better biological clarity and clinical interpretability, but may miss broader disease-related molecular information. In contrast, label-free SERS fingerprints capture richer molecular signatures and support multi-class discrimination, although biological interpretation and reproducibility remain major challenges. Among the other studies, the ML-driven microfluidic deglycosylation chip for PD-L1 EVs detection [57] appears particularly promising because it addresses a clinically relevant problem related to unreliable PD-L1 assessment for cancer immunotherapy. However, direct comparison among studies remains difficult because different biomarkers, sample types, AI models, and performance metrics are used. CNN-, ResNet-, YOLO-, RF-, and MLP-based models can improve automated image or spectral analysis. However, many reports are limited by small cohorts, retrospective datasets, and cell-line samples, limiting confidence in clinical generalization. In addition, diagnostic cost may increase due to microfluidic fabrication, nanomaterials, optical instrumentation, exosome isolation, and sample preparation costs. Therefore, future cancer biochip studies should include large multicenter clinical validation and standardized reporting of sensitivity, specificity, AUC, response time, TAT, sample type, diagnostic cost, dataset size, and external validation before clinical translation.

#### 2.1.3. Diabetes Diagnosis

**Figure 4:**
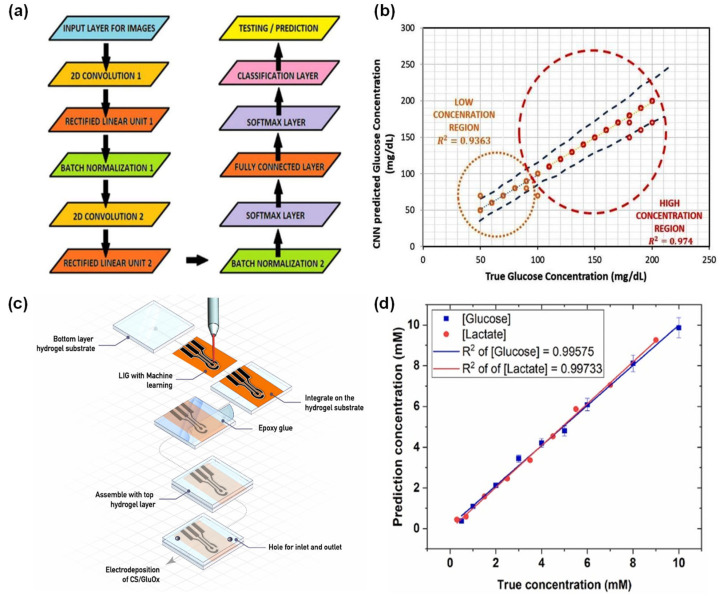
(**a**) Schematic of CNN-based image classification pipeline with convolution, rectified linear unit layer, batch normalization, and fully connected SoftMax layers, reprinted with permission from [60], copyright CC BY 4.0. (**b**) CNN prediction performance for glucose detection with validation of ISO 1597:2013/2015 chip, reprinted with permission from [60], copyright CC BY 4.0. (**c**) Fabrication of a flexible sodium alginate hydrogel microfluidic chip with glucose oxidase-functionalized LIG electrode, reprinted with permission from [61], copyright 2025 Elsevier. (**d**) RF regression showing strong agreement between predicted and actual concentration, reprinted with permission from [61], copyright 2025 Elsevier.

Diabetes is a long-term disease that affects the body regulation of blood sugar, leading to hyperglycemia, and it is a major global health concern daily monitoring plays a significant role in ensuring effective treatment. For daily monitoring, AI-integrated biochips are gaining momentum in diabetes monitoring because they can bring glucose analysis closer to real-time, low-cost, and potentially non-invasive care. AI-integrated biochips can manage complex biological signals more consistently than manual interpretation alone. ML and DL models are used to classify diabetes monitoring, but also as a tool for signal interpretation, compensating for variability, automating readout, and sensor fabrication. For example, Kanchan et al. (2024) [60] reported a smartphone-based colorimetric platform, which developed a multilayer polyvinyl film microfluidic device for glucose monitoring through glucose oxidase/peroxidase reaction and image analysis by CNN models. The purpose of the platform is to create an affordable POCT platform that performs reliable glucose detection with minimal instrumentation and without dependence on conventional calibration-heavy readout systems. The biochips consisted of stacked adhesive-coated polyvinyl chloride film on a PMMA base, with capillary-derived channels and defined inlet and outlet wells. The sample set covered prepared glucose solutions from 50 to 200 mg/dL, and the resulting color change was captured under different lighting conditions and with different smartphones. The integrated CNN colorimetric sensor system demonstrates a 95% overall accuracy in detecting glucose within novel images, with precision, recall, and F1 scores being 94, 93, and 93%, respectively, and predictions for both low and high concentrations exhibited robust concordance with the International Organization for Standardization (ISO)-based evaluation standards. This research is innovative in its integration of a straightforward plastic microfluidic colorimetric chip with smartphone imaging and CNN classification, designed for user applications. The main benefits are its portability, easy preparation, its practical design for use in POCT, and its ability to produce consistent images. However, this study clinical applicability remains incomplete, as it was conducted using prepared glucose samples rather than actual patient samples (Figure 4a,b) (Table 3) [60].

Ambia et al. (2025) [61] reported advanced the field of flexible continuous monitoring by creating a smart microfluidic biosensor. This biosensor was designed with a laser-induced graphene (LIG) electrode, which was modified using chitosan-functionalized glucose oxidase and crosslinked with polyethylene glycol. The main goals of this platform were to prepare an affordable microfluidic system that could reliably detect glucose, while also improving its durability, ease of manufacturing, and ability to differentiate analytes in real-time within complex environments. The platform combined a soft microfluidic structure with ML-optimized LIG electrodes and enzymatic electrochemical sensing. The RF regression was used to optimize LIG fabrication parameters, reaching an accuracy of around 90% and cutting fabrication time by 60%. The RF-optimized sensor achieved glucose response from 0.4 to 10 mM with sensitivities of 18.0 µA in the flat state and 17.6 µA under bending. Also, ML-based signal deconvolution enables simultaneous real-time quantification of glucose and lactate in mixed samples, with root-mean-square error (RMSE) values of 0.18 mM and 0.21 mM, respectively. The study findings indicate good mechanical durability, with over 90% of the signal remaining after voltammetry cycles at 45 °C and bending. This suggests the potential for using this technology in continuous glucose monitoring. However, the absence of validation in clinical human samples indicating translational applicability has not yet been fully stabilized (Figure 4c,d) [61]. Kaliappan et al. (2026) [62] introduced a new method suggesting a non-invasive glucose monitoring system based on RF sensing. This system used an octagonal complementary split-ring resonator, arranged in a honeycomb pattern, and functioned at a frequency of 2.45 GHz. The primary objective of this platform was to develop a painless biosensor capable of detecting glucose-related dielectric alterations. Furthermore, biochip platform aimed to achieve enhanced performance and a more straightforward signal-processing methodology compared to more complex AI models. The RF microwave biosensor was evaluated for its ability to assess glucose concentrations ranging from 0 to 300 mg/dL, employing electromagnetic analysis of the reflection coefficient. Among the models evaluated, the CNN demonstrated the highest accuracy, achieving 96.8%, followed by the SVM at 95.1%. The singular value decomposition (SVD)-based method, however, attained an accuracy of 94.2%, despite its considerably lower model complexity, a training time of 0.42 s, and an inference time of 3.5 ms. Furthermore, the study documented consistent sensing performance, repeatable measurements, and the method applicability to thin packaging layers up to 1 mm thickness without significant signal degradation. The primary innovation lies in the combination of a compact octagonal-shaped complementary split-ring resonator sensor with lightweight ML models for wearable applications. Nevertheless, its assessment has been limited controlled glucose environments, and clinical validations using real samples have not yet been reported (Table 3) [62].

Overall, the AI-integrated biochips show strong practical value for diabetes monitoring because glucose detection requires rapid, low-cost, repeated, and reliable measurement. The reported platforms include smartphone-assisted colorimetric chips, flexible electrochemical microfluidic sensors, RF/microwave-based non-invasive sensors, and ML-optimized electrode systems. Smartphone colorimetric biochips are simple, portable, and low-cost, but their accuracy can be affected by lighting conditions, camera variation, image quality, and sample matrix effects. Flexible electrochemical microfluidic chips are more suitable for wearable and continuous monitoring. However, long-term enzyme stability, electrode fouling, mechanical deformation, calibration drift, and reproducible fabrication remain important limitations. RF/microwave-based non-invasive sensors avoid blood sampling and improve user comfort, but their performance can be influenced by body movement, tissue variability, temperature, hydration level, and environmental interferences. AI models such as CNN, RF, SVD, and RF have been used for image classification, signal classification, lightweight RF signal interpretation, and manufacturing optimization, respectively. Among the reported studies, the ML-optimized laser-induced graphene electrode-based smart microfluidic biosensor [61] appears particularly promising because it integrates flexible microfluidics, enzymatic glucose sensing, machine learning-guided fabrication, mechanical durability, and real-time discrimination of glucose from lactate in mixed samples. This combinations are important for practical diabetes monitoring because wearable systems require not only high sensitivity, also mechanical robustness, reproducible manufacturing, stable calibration, and resilience to signal overlap in complex physiological samples. However, most reported systems still require validation using larger human datasets and real daily user conditions. Therefore, future studies should focus on long-term operational stability, inter-device calibration, real-sample testing, user cost, portable hardware integration, response time, TAT, and clinically accepted accuracy standards before translation into practical diabetes-monitoring devices.

### 2.2. AI-Integrated Biochips for Diagnosis and Monitoring of Neurological Disorders

AI-integrated biochips are emerging as a promising platform for the diagnosis and monitoring of neurological disorders, because these diseases are biologically complex, clinically heterogeneous, and often difficult to detect early with routine methods. Their main advantages are enhanced different layers of neurological diagnostics, from pattern recognition in molecular and spectral data to risk prediction from longitudinal clinical records. Reported studies have demonstrated their diversity, including fluorescent sensor arrays, metabolomics pipelines, infrared metasurfaces, electronic health record models, SERS substrates, graphene field-effect transistor biosensors, microfluidic electrophysiology systems, and saliva-based microfluidic immunoassays. In this section, we discuss AI-integrated biochips applied to the diagnosis and monitoring of neurological disorders. Kavungal et al. (2023) [63] introduced a sensor that combined an immunoassay with a nanoplasmonic infrared metasurface. This sensor was designed to detect a biomarker linked to neurodegenerative diseases. The microfluidic immuno-surface-enhanced infra-red absorption (SEIRA) platform was engineered to capture misfolded proteins, such as α-synuclein and to distinguish between oligomeric and fibrillary forms by analyzing their infrared absorbance patterns. Furthermore, an ANN was employed to quantify the proportions of mixed aggregate species. The biochip design allowed for multiplexing using different sensing elements that were specifically designed to detect α-synuclein and tau proteins. The key contribution of this platform does not merely measure protein abundance, but it also attempts to read structural state, which is a more disease-relevant property in many neurodegenerative conditions. The platform shows strong potential by combining microfluidics, plasmonic enhancement, immunocapture, and AI to detect pathological signatures from human cerebrospinal fluid, but its broader application is limited by the need for further clinical validation in complex biological samples [63]. Kim et al. (2024) [64] implemented a surface-functionalized SERS platform with DL for label-free blood-based Alzheimer diagnosis. Gold nanowire arrays were prepared in two functional forms, and one of which used the 6E10 antibody to capture Aβ(1–42) and analyze oligomerization, while the other used self-assembled monolayers to interact with blood-based metabolites. Human plasma from Alzheimer’s patients and healthy controls was then measured, and the spectra were analyzed with a fully connected neural network. The study reported that plasma classification on the antibody substrate reached 87.5% accuracy with a sensitivity of 82.9% and specificity of 92.2%, while metabolite-based analyses reached accuracies up to 99.5%. Explainable AI (XAI) was also employed to pinpoint the spectral regions that influenced classification outcomes. A primary benefit is the capacity for direct comparative analysis, which reveals that metabolite-based SERS signals offer superior diagnostic value in plasma samples. Conversely, a notable constraint arises from the reliance on preprocessing methods, substrate uniformity, and the model’s generalizability, thus requiring more comprehensive validation (Table 4) [64].

**Figure 5:**
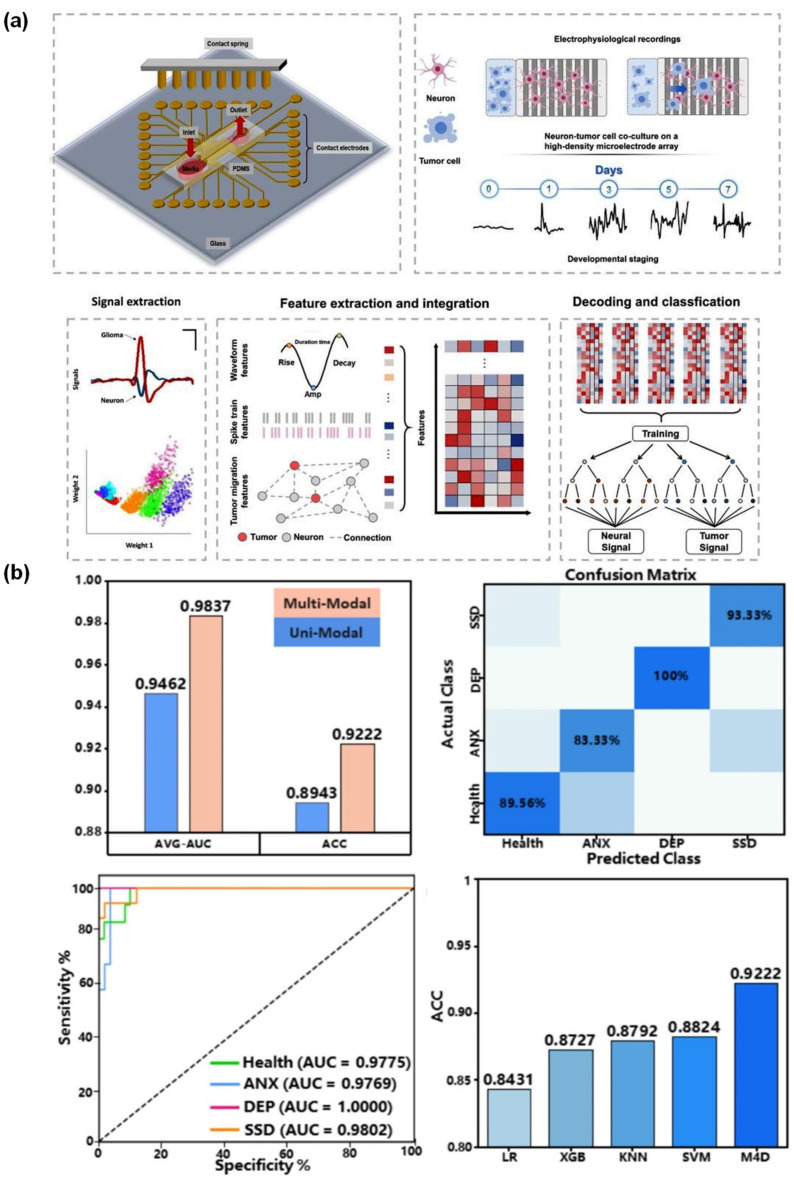
(**a**) Schematic of the microfluidic MEA system showing neuron-tumor co-culture electrophysiological recording and the integrated workflow for signal extraction, feature analysis, and classification, reprinted with permission from [66], copyright CC BY 4.0. (**b**) Overview and performance of the multimodal MEA-based system for neuron–tumor interaction analysis and mental disorder classification, including device design and evaluation metrics (AUC, accuracy, receiver operating characteristic curve (ROC), and confusion matrix), reprinted with permission from [67], copyright 2026 Elsevier.

Wang et al. (2024) [65] presented a novel report that detailed the creation of an extremely sensitive, multi-channel graphene field-effect transistor biosensor. This platform was designed for screening Alzheimer’s disease using plasma samples. The device included five microfluidic detection channels and was able to measure femtomolar concentrations of Aβ40, Aβ42, P-tau181, P-tau217, and NfL in multicenter clinical cohorts. The RF and SVM models integrated and then combined plasma biomarkers with clinical information to build a “composite-info” panel. This approach achieved AUC values around 0.94 and showed better staging performance for normal controls, mild cognitive impairment, and Alzheimer’s disease compared with single biomarkers and magnetic resonance imaging-based assessment in the reported study. The system offers a minimally invasive, highly sensitive, and multiplexed detection method. It combines label-free transistor sensing with machine learning analysis of multiple biomarkers. However, the clinical use of this method is limited by the small number of participants in the study and the need for more thorough testing [65]. Xu et al. (2026) [66] reported that the electrophysiological microfluidic biochips could diagnose Alzheimer’s disease with 83% accuracy. The authors developed a customized microfluidic electrophysiological system incorporating microelectrode arrays (MEA), stimulation hardware, signal-processing layers, and LR-, RF-, and MLP-based modeling to elucidate neural-glioma communication. The sensor system was trained on 127,000 signals and used to find patterns in the signals that were linked to glioma cell invasion and hyper-invasive behavior when neurons were present. This platform is new because it treats neural electrical activity as a functional biomarker space and combines monitoring with intervention. The platform provides robust integration of various advanced methodologies for mechanistic neuro-oncology research. However, these platform dependence on in vitro interaction analysis constrains its immediate applicability in clinical diagnostics and indicates a protracted trajectory towards real-world implementation (Figure 5a) (Table 4) [66].

The RF integration of a microfluidic chip by Wang et al. In 2026, a study [67] enabled more precise multiplex sensing for conditions such as anxiety, depression, and somatic symptom disorder (SSD). This platform used microfluidic chips to assess nine salivary biomarkers in a cohort of 300 participants. This cohort included both healthy individuals and those diagnosed with depression, anxiety, and SSD. A model called M4D was then created using a smaller set of four biomarkers: TNF-α, IL-8, IL-1β, and IFN-γ. This model showed AUC values above 0.98 and a test accuracy of 92.22%. The study suggests saliva as a less invasive alternative to blood sampling, a particularly pertinent consideration for ongoing mental health evaluation. This method has some benefits, such as that it uses non-invasive sampling, uses a relatively large dataset, and it combines biomarker, clinical, and demographic data into a model that is easy to use. Limitations include its pre-proof status, the fact that biomarkers are variable across studies, the possible dataset bias and overfitting, and the need for external validation and standardized panels (Figure 5b) [67].

AI-integrated biochips for neurological disorder diagnosis are promising because they can detect low-abundance biomarkers and analyze complex molecular patterns that are difficult to interpret using conventional methods. The reported platforms include synthetic fluorescence arrays, omics-based pipelines, infrared, and Raman nanoplasmonic sensors, graphene transistor biosensors, microfluidic immunoassays, electrophysiological chips, and virtual platforms based on electronic health records. These systems have been combined with different AI approaches, including DLA, RF, multilayer perceptron, ANN-based spectral quantification, and XAI modules. Among the reported studies, the graphene field-effect transistor platform [66] appears particularly promising for neurological diagnosis and monitoring because it combines minimally invasive plasma sampling, femtomolar sensitivity, multiplexed detection of established Alzheimer’s disease biomarkers, microfluidic integration, and ML-based stage discrimination. The integrated design is important because it performed better than single-biomarker analysis and showed comparability with common clinical assessments. However, neurological biochips have stronger translational barriers than pathogen- or glucose-detection platforms. Many neurological biomarkers show large patient-to-patient variation, and some clinically relevant samples, such as cerebrospinal fluid, are invasive to collect. Although SERS, infrared, graphene transistor, and microfluidic immunoassay platforms can provide high performance, they often require complex fabrication, expensive instrumentation, strict signal normalization, and carefully controlled sample processing. Direct comparison of studies is also difficult because of the different biomarker panels, sample types, AI models, sensitivity values, and clinical endpoints used. In addition, many AI models may be biased because they are trained using limited clinical cohorts, signal-center datasets, and selected biomarker panels. Therefore, future studies should focus on multicenter validation, standardized biomarker panels, transparent and XAI models, reproducible device manufacturing, response time, TAT, and cost-effective platform design for reliable neurological diagnosis.

### 2.3. AI-Integrated Biochips for Drug Delivery and Diagnosis

AI-integrated biochips are becoming increasingly important for drug delivery, drug development, and drug testing. Because, they help fill a long-standing gap between simple in vitro tests and the more complex biological conditions that affect drugs work in vivo. The AI is built to find new drug candidates from big biological datasets and then test them in POCT systems. Micro physiological platforms also produce high-content imaging data that ML can use to classify drugs, score their effectiveness, and predict their toxicity. Another direction is the creation of physiologically enhanced POCT chips for absorption studies, where the chip itself enhances biological relevance even AI is not the main analytical layer yet. In general, biochips that use AI are moving preclinical testing away from isolated endpoint assays and toward workflows that are more integrated, measurable, and mechanistically informative. In this section, we discuss AI-integrated biochips that were applied to drug delivery, drug development, and drug testing. Chong et al. (2022) [68] reported a major problem in systemic drug development, the lack of a human-relevant platform to prospectively predict skin-sensitizing liability before clinical exposure. A microfluidic multicellular co-culture array with four connected compartments containing hepatocyte spheroids, antigen-presenting cells, keratinocytes, and dermal fibroblasts, allowing drug metabolism and downstream immune and dermal responses to be represented in a single platform. The microfluidic biochip was integrated with PCA and an SVM classifier to identify sensitizing and non-sensitizing drugs. The SVM and PCA models achieved significantly higher detection accuracy of 87.5% compared with a specificity of 75% and a sensitivity of 100% under stratified four-fold cross-validation. The AI-integrated biochips were used prospectively for obeticholic acid, where the combined chip and ML workflow predicted cutaneous sensitization and suggested FasL-mediated keratinocyte apoptosis rather than immune activation as the dominant mechanism. A key advantage is its multicellular, mechanism-aware ML screening design with translational relevance and high-content compatibility, while its main limitation is the small training dataset, which reduces specificity and limits generalizability [68]. Samantasinghar et al. [69] focused on AI-integrated drug repurposing for tubule-interstitial fibrosis, a major driver of chronic kidney disease. The ML-based repurposing framework SperoPredictor V1.2 was used together with downstream literature review and molecular docking to identify candidate-approved drugs, from which lubiprostone emerged as the best-performing option for validation. The biochip-proximal tubule-on-a-chip system was designed as a more physiologically relevant renal fibrosis model than conventional 2D culture, and the selected drug was also validated in a unilateral ureteral obstruction (UUO) mouse model. Lubiprostone significantly reduced connective tissue growth factor, fibronectin, collagen, Smad2/3, MMP2/9, PAI-1, EMT-related markers, and JAK/STAT3 signaling in both chip and in vivo models, suggesting multi-pathway anti-fibrotic action. Biochips combine AI-based drug repurposing with mechanistic organ-chip validation in a more in vivo-like preclinical model; limitation: the study mainly validated one shortlisted drug, so broad benchmarking across multiple candidates was limited (Table 4) [69].

**Figure 6:**
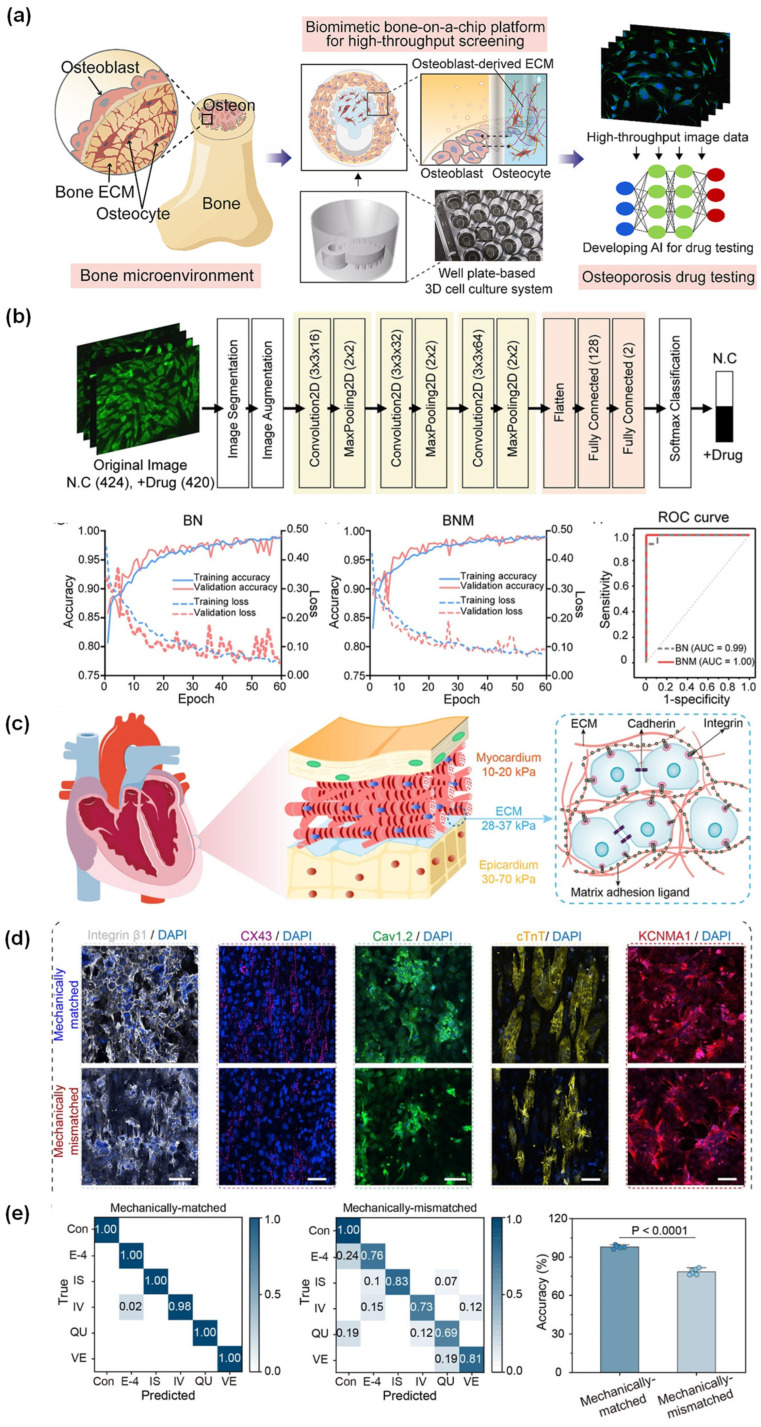
(**a**) Schematic overview of a biomimetic bone-on-a-chip platform combined with AI-integrated image analysis for high-throughput drug testing, reprinted with permission from [70], copyright CC BY 4.0. (**b**) CNN-based DL model for osteoporosis drug testing, showing training and validation accuracy-loss trend for BN and BNM datasets across epochs, ROC, and AUC, reprinted with permission from [70], copyright CC BY 4.0. (**c**) Schematic showing cardiomyocytes growing on a bilayer flexible substrate in vivo, reprinted with permission from [72], copyright 2025 ACS. (**d**) Representative immunofluorescence images of integrin β1, CX43, Cav1.2, cTnT, and KCNMA1, reprinted with permission from [72], copyright 2025 ACS. (**e**) Heart-on-chip classification performance comparing mechanical matching and mismatch conditions, reprinted with permission from [72], copyright 2025 ACS.

Paek et al. (2023) [70] developed a bone-on-a-chip paper targeted at osteoporosis drug testing and aimed to produce a more realistic and high-throughput bone microenvironment for preclinical evaluation. The authors fabricate a well plate-based biomimetic bone-on-a-chip in which osteocyte-like cells and osteoblasts were co-cultured within an osteoblast-derived decellularized extracellular matrix, with the chip geometry designed to mimic the osteon and support cell-cell interaction. The CNN-based image analysis system was used to evaluate anti-SOST antibody treatment through β-catenin fluorescence and nuclear translocation patterns. The platform generated extensive image datasets suitable for high-content screening, and the DL classifier demonstrated a 97.2% accuracy rate, with an AUC of 0.99, when utilizing β-catenin and nucleus images. Furthermore, an accuracy of 99.5% and an AUC of 1.00 were achieved with merged images. A key advantage limitations in the capacity to integrate a biomimetic bone chip with AI-driven image analysis, thereby facilitating the rapid and precise evaluation of biologically pertinent drugs. Conversely, a significant limitation is the absence of osteoclasts, which constrains the comprehensive modeling of osteoporosis and the broader assessment of drug responses (Figure 6a,b) [70]. Sun et al. (2025) [71] reported that CPHNet research moved drug discovery toward an image-first phenotypic strategy. This platform discovers therapeutic agents for high-altitude pulmonary edema by identifying compounds that reverse hypoxia-induced morphological changes in alveolar epithelial and pulmonary microvascular endothelial cells. The workflow started with Cell Painting imaging, which produced more than 100,000 full-field images and more than 200,000 images of subcellular structures. The main AI pipeline was made up of two DL models: SegNet for segmenting and HypoNet for scoring hypoxia. The candidate compounds identified through this framework were subsequently validated using a 3D alveolus chip model and in murine subjects, resulting in ferulic acid and resveratrol emerging as the most promising anti-HAPE agents. The study emphasizes a novel biochip that incorporates high-content morphological phenotyping, DL, and organ-on-chip validation. The main thing about it is that the AI can turn complicated cellular morphology into a scalable drug-screening score.

On the other hand, the alveolus chip provide physiological relevance that goes beyond traditional 2D cultures. For example, they only used controlled laboratory settings, the quality of the training data for DL models and A549 cancer-derived epithelial cells instead of primary alveolar cells, which could make their work less useful in other situations [71]. Han et al. (2025) [72] created a heart-on-a-chip platform to mitigate mechanical discrepancies between conventional materials and native myocardial tissue, thereby improving both excitation-contraction coupling and the precision of drug testing. They employed porous polydimethylsiloxane (PDMS) to fabricate a substrate exhibiting a Young’s modulus more closely aligned with that of epicardial and extracellular matrix tissue. This system successfully recorded electromechanical behavior over an 11-day period and endured more than 1,000,000 stretch cycles, resulting in a 128% enhancement in excitation-contraction coupling. AI-integrated biochips and organ-on-chip systems provide valuable platforms for drug screening, toxicity testing, disease modeling, efficacy testing, transport analysis, and drug response prediction. Compared with conventional 2D cell culture systems, these platforms better mimic physiological microenvironments and generate high-content imaging, mechanical, molecular, and functional data suitable for AI-assisted analysis (Figure 6c,d,e) [72]. In the reported studies, AI was applied for early-stage drug selection, phenotypic evaluation, image-based analysis, and integration of multiple chip-derived signals. For example, ML-assisted drug classification linked mechanical biomimicry with the quality of pharmacological readouts, highlighting the potential of biochips for more realistic drug evaluation. Among the reported platforms, the multicellular cutaneous-reaction platform [68] appears important because it integrated liver, immune, and skin responses with ML-based toxicity prediction, making preclinical testing more biologically relevant and more informative for drug safety assessment. However, several important limitations remain, for example studies lack detailed classifier performance metrics, drug panel information, and comprehensive pharmacological validation. In many cases, AI models were trained using controlled laboratory datasets, and only a limited number of candidate drugs were experimentally validated. In addition, the broader clinical and industrial translation of these systems is limited by complex chip fabrication, high experimental cost, small training datasets, and a lack of standardized drug testing protocols. Organ-on-chip systems require stable cell culture conditions, long-term reproducibility, and stronger correlation with in vivo pharmacokinetic and pharmacodynamic responses. Therefore, future studies should focus on large drug panels, stronger biological validation, cost assessment, standardized performance reporting, reproducible chip fabrication, and more reliable integration of AI with chip-derived datasets.

## 3. Conclusions and Challenges

This review provides a comprehensive view of the development of AI-integrated biochip platforms for rapid and accurate on-site diagnosis of infectious diseases, cancer, diabetes, neurological disorders, and drug delivery and monitoring. Conventional biochip platforms, primarily microfluidic and LOC chips, have emerged as effective tools for POCT diagnostics due to their rapid processing, simple operation, and low reagent consumption while mimicking physiological microenvironments. The integration of AI with biochip systems has significantly improved diagnosis performance by enabling automated analysis of complex signals from optical, colorimetric, fluorescence imaging, spectroscopic, SERS, and electrochemical-based platforms. DL, ML, and RL models enhance feature extraction, noise filtering, and pattern recognition, allowing detection of pathogens, cancer biomarkers, neurological disorders, diabetes, and drug delivery and monitoring. In this review, we explored recent AI models for disease diagnostic accuracy and drug monitoring, including SVM, RF, DT, LR, PCA, CNN, ResNet, YOLO, SegNet, HypoNet, MLP, ANN, DFFNN, and SNN. These AI models have been analyzed for color patterns, spectral noise, viscosity, and images. Data preprocessing strongly affects the performance of AI-integrated biochip systems. Proper denoising, normalization, baseline correction, background subtraction, and feature extraction can reduce experimental variations and improve diagnostic accuracy. However, the lack of standardized preprocessing and evaluation protocols remains a major challenge, making it difficult to compare AI model performance across different biochip platforms. AI approaches enable multiplex detection, especially of diabetes and pathogens such as *E. coli*, *S. aureus*, *S. typhimurium*, *E. faecium*, *P. aeruginosa*, *L. monocytogenes*, *Salmonella*, *B. cereus*, HPV-16, and HPV-18. Also, AI is used to effectively find neurological diseases, such as Alzheimer’s disease, depression, and anxiety, and cancer, following lung, breast, colon, liver, pancreatic, stomach, and exosome-related tumors.

However, several biochip-specific and diagnostic concerns remain unresolved. Although AI-assisted biochips have improved sensitivity, accuracy, and response time, several issues still limit their clinical translation. Performance metrics are inconsistently reported across studies, with some focusing on accuracy and others reporting LOD, AUC, TAT, sensitivity, specificity, and response time. Many AI models are also trained using small, controlled, single-center datasets, which increases the risk of overfitting, selection bias, and poor generalization to real clinical samples. Moreover, diagnostic cost and TAT are often not clearly reported, despite being essential for POCT applications. In addition to analytical limitations, diagnostic TAT and test cost remain major challenges for routine clinical diagnosis, as conventional methods such as culture, AST, PCR, histopathology, imaging, LC-MS, and ELISA can require hours to days and cost from a few dollars to several hundred dollars per test. Therefore, future AI-integrated biochip platforms should reduce both TAT and per-test cost by minimizing reagent use, sample-processing steps, instrumentation dependence, and skilled-labor requirements for affordable POCT diagnostics.

Conventional biochips are often non-specific, prone to signal fluctuation, and have limited sensitivity for low-abundance biomarkers, which directly affect diagnostic accuracy. AI-integrated platforms have issues because they do not autonomously retrain or update controlled datasets, have lower diversity, and are pre-trained on small datasets, limiting model generalization to real clinical samples. The lack of standardized protocols for chip fabrication, fluid handling, and sensing probes introduces batch-to-batch inconsistencies, and camera models and image acquisition conditions significantly reduce reproducibility across platforms. Moreover, the lack of standardized data acquisition, preprocessing, and evaluation protocols poses significant challenges and difficulties in comparing model performance. In addition, many AI models remain non-interpretable, which also reduce ratability of analytical performance. High computation demand and energy consumption restrict the development of AI-integrated biochips in portable and resource-limited areas. To address these limitations, future research is required to use diverse, larger, and clinically relevant datasets to improve model robustness. Standardization of AI-integrated biochip fabrication, experimental processing, and data processing workflow is essential to ensure reproducibility and stability. Furthermore, integrating XAI will improve interpretability, reduce energy consumption, and support clinical analysis. Therefore, future studies should focus on large drug panels, stronger biological validation, cost assessment, standardized performance reporting, reproducible chip fabrication, and more reliable integration of AI with chip-derived data.

## 4. Future Perspectives

Future development of AI-integrated biochips should move beyond proof-of-concept demonstrations toward clinically validated and standardized diagnostic platforms. Large multicenter datasets, external validation, and prospective clinical testing are required to confirm model robustness and reduce overfitting or dataset bias. Lightweight and explainable models should be integrated with smartphone-based or portable biochip systems to reduce computational cost and improve user trust. In addition, future studies should clearly report diagnostic cost, assay time, reagent stability, and device reproducibility to support practical translation. Future AI-integrated biochip platforms should focus on low-cost chip materials, reduced reagent consumption, automated sample preparation, smartphone-compatible readout, lightweight AI models, and minimal instrument dependence to achieve rapid, scalable, and clinically affordable POCT diagnostics. Combining standardized biochip fabrication, automated sample preparation, cloud AI analysis, and interpretable decision support will be essential for developing reliable next-generation POCT systems. Near-future studies should prioritize the development of lightweight and computationally efficient AI models for integration with portable and smartphone-based biochip systems, enabling real-time POCT diagnostics. In parallel, standardization of imaging conditions, sensor configuration, and signal acquisition protocols must be established to minimize variability. Future AI-integrated biochip studies should establish standardized preprocessing workflows, including data cleaning, denoising, normalization, baseline corrections, and feature extraction. Such standardization will improve reproducibility, allow fair comparison between models, and support clinical translation. In addition, lightweight AI models combined with XAI and validated preprocessing pipelines will be important for portable and reliable POCT biochip systems. Optimization of AI-integrated biochips through energy-efficient processors, online learning adaptation, and hardware accelerators ensures fair competition (data cleaning and noise removal, data normalization, data formatting, and organization) and will further enhance system feasibility, particularly in low-resource areas. Future studies should standardize camera models and image acquisition conditions to significantly reduce reproducibility across platforms. In the long-term goal, AI-integrated biochips are expected to evolve into autonomous and adaptive diagnostic systems capable of continuous learning and dynamic optimization of sensing conditions. To move these platforms from laboratory studies to practical use, it is necessary to plan development, validation, and implementation in multiple environments. In the early stage, priority should be given to establishing clear validation procedures and consistent performance metrics so that results can be compared to overall studies. At the time, the development of regulatory guidelines and consensus frameworks will be important to ensure reliability and support future innovation.

Overall, addressing these near-term and long-term directions will help transform AI-integrated biochips through collaboration between materials scientists, engineers, clinicians, and data scientists. The key aspects of XAI methods can help explain decision-making procedures and increase confidence among end users. Therefore, overcoming current limitations through collaborative and standardized research will be crucial for the practical use of AI-integrated biochip systems.

## Figures and Tables

**Table 1:** Conventional diagnostic methods and approximate TAT.

Sl. No.	Application	Method	Approx. TAT	Approx. Cost Consideration (USD)	Limitation
1	Bacterial infection	Culture	24–72 h	10–40	Slow growth
2	AMR	Culture + AST	24–48 h	20–60	Delayed therapy
3	Viral infection	PCR/RT-PCR	1–4 h	50–150	Need instruments
4	Cancer	Histopathology/molecular profiling	Hours to days	100–500	Specialist interpretation
5	Diabetes	Glucose meter/HbA1c	Second to hours	1–2	Lab dependence for HbA1C
6	Neurological disorders	Imaging/immunoassays	Hours to days	50–1000	Costly and complex
7	Drug monitoring	HPLC/LC-MS/ELISA	Hours to days	60–300	Centralized instruments

**Table 2:** Overview of ML, DL, and RL algorithms.

Category	Learning Type	AI Algorithm	Working Principle	Input Data Type	Data Requirements	**Practical Considerations**
Machine learning (ML)	Supervised learning	Support vector machine (SVM)	Constructs an optimal separating hyperplane that maximizes the margin between different classes	Handcrafted or extracted features	Labeled data required	Sensitive to kernel selection and parameter tuning
		Random forest (RF)	Builds multiple decision trees using random subset of data and features, and combines their outputs for classification or regression	Structured, tabular, or extracted features	Labeled data required	Robust to noise but may overlook subtle anomalies
		k-Nearest neighbor (kNN)	Classifies a new sample based on the majority class of its nearest labeled neighbors in the feature space	Structured, or extracted features	Labeled data required	Computationally expensive for large datasets; sensitive to scaling
		Linear discriminant analysis (LDA)	Finds linear combinations of features that maximize class separation while minimizing within-class variance	Structured, or extracted features	Labeled data required	Limited to linear class separation
		Artificial neural network (ANN)	Learns nonlinear relationships between input features and output labels through interconnected weighted neurons	Structured, or extracted features	Labeled data required	Prone to overfitting; requires careful optimization
	Unsupervised learning	k-means	Partitions unlabeled data into k clusters by minimizing the distance between data points and their assigned cluster centroids	Structured, extracted features, or raw data	Unlabeled dataset	Sensitive to initial cluster selection
		Principal component analysis (PCA)	Reduces data dimensionality by transforming correlated variables into orthogonal principal components that retain maximum variance	Structured, extracted features, or image-derived features	Unlabeled dataset	May lose important information
		Self-training	Uses a model trained on labeled data to generate pseudo-labels for unlabeled data and iteratively improve learning	Raw or feature data	Small labeled dataset	Risk of error propagation
		Graph-based method	Constructs a similarity graph and propagates label information from labeled samples to unlabeled samples	Feature data or graph-structured data	Small labeled dataset	Computational complexity increases with dataset size
Deep learning (DL)	Supervised learning	Convolutional neural network (CNN)	Automatically extracts spatial and hierarchical features from image data using convolutional and pooling layers	Raw images or image-like data	Large labeled image dataset	Requires GPU; high computational cost
		You only look once (YOLO)	Performs real-time object detection by predicting bounding boxes and class probabilities in a single forward pass	Raw images	Annotated images with bounding boxes	Fast but less accurate for dense objects
		U-shaped network (U-Net)	Uses an encoder–decoder architecture with skip connections for accurate pixel-level image segmentation	Raw images	Pixel-wise annotated datasets	High accuracy but computationally intensive
		Residual network (ResNet)	Uses residual or skip connections to improve gradient flow and enable the training of very deep neural networks	Raw images or image-like data	Labeled image dataset	High accuracy; expensive training
		MobileNet	Uses lightweight depth-wise separable convolutions to reduce computational cost for mobile and edge-based image analysis	Raw images	Labeled image dataset	Fast and efficient; reduced accuracy
	Semi-supervised learning	Variational auto encoders (VAE)	Learns a probabilistic latent representation of input data using an encoder–decoder framework for reconstruction and generation	Raw images or high-dimensional data	Mostly unlabeled dataset	Useful for denoising and feature extraction
Reinforcement learning (RL)	RL	Q-learning/Deep Q-Network (DQN)	Learns optimal actions through reward-based interaction; DQN use deep neural networks to approximate action-value functions	Raw images and sensor data	Interaction with an environmental function	High computational cost; complex design

**Table 3:** Overview of AI-integrated biochips for disease diagnosis, such as pathogens, cancers, and diabetes.

Sl. No.	AI Algorithm	Platform/Biochips Type	Target Disease	Sensor Method	Performance	Advantages	Limitations	Ref.
1	SVM	Smartphone-based paper microfluidic chip	*E. coli*, *S. aureus*, *S. typhimurium*, *E. faecium*, and *P. aeruginosa*	Flow velocity patterns	Accuracy: 93.3% and response: <10 min	Portable, low-cost, and field deployable	Limited to trained dataset	[43]
2	SNN	SERS nanowire chip	*AMR E. coli*	Raman spectral fingerprint	LOD: 100 CFU/mL	AMR detection	Complex fabrication	[44]
3	PCA	Lab-built fluorescence spectrometer	*S. aureus*, methicillin-resistant *S. aureus*, *E. coli O157:H7*, and *E. coli ER2738*	Fluorescence patterns	Accuracy: 100% and response: 120 min	High specificity	Long incubation time	[45]
4	DFFNN	Paper chromogenic array sensor	*L. monocytogenes*, *Salmonella*, and *E. coli O157:H7*	Color patterns	Accuracy: >90% and LOD: ~1 log CFU/g	Non-contact detection, multiplex sensing, and portable	Time-dependent response	[46]
5	SVM	Liquid crystal optical sensor array	*B. cereus*, *E. coli*, *P. aeruginosa*, *S. aureus*, and *S. typhimurium*	Optical patterns	Accuracy: >98.89% and LOD: 10 CFU/mL	Low LOD and low-cost	Optical instrument needed	[47]
6	ResNet-18 (CNN)	Microfluidic fluorescence biosensor	*E. coli*	Fluorescence patterns	Accuracy: 99%, LOD: 2 CFU/mL, and response: 90 min	Low LOD and integrated enrichment	Long incubation time	[48]
7	DNN	Microfluidic chip	*Mycobacterium tuberculosis* (*M. bovis*)	Phase-contrast growth in microscopy	Accuracy: 99.96% and pDST: 12 h	Rapid TB phenotypic DST	Need real TB sample validation	[49]
8	ResNet-18	Microfluidic blood bacteria detection chip	Sepsis (*E. coli*, *K. pneumoniae*, and *E. faecalis*)	Microscopic imaging pattern	LOD: 1 to 10 CFU/mL and response: 2 h	Culture-free and low LOD	*S. aureus* detection remains challenging	[50]
9	Python-based image analyzer	Droplet microfluidic chip	Heteroresistant *E. coli* bloodstream infection	Droplet shrinkage patterns	Detect resistant subpopulations 10^−6^ and response: 12 to 24 h	Detect rare antibiotic-resistant subpopulations	Longer incubation and droplet setup required	[51]
10	ResNet-18 (CNN)	Hand-driven microfluidic CRISPR chip	*HPV-16* and *HPV-18*	Fluorescence patterns	Accuracy: 95%, LOD: 10^−18^ M, response: 60 min	Low LOD and low-cost	Long incubation time	[52]
11	DT, RF, and SVM	Magnetic nanowaxberry chip	Lung cancer	Fluorescence patterns	Accuracy: 96%	Simultaneous detection	Requires complex probe design	[53]
12	PCA	ExoSIC-magnetic nanowaxberry chip	Lung cancer	Fluorescence patterns	AUC: 0.791	High purity	Limited multiplex detection	[54]
13	CNN, SVM, MLP	Exosome–SERS–AI biochip	Lung, breast, colon, liver, pancreas, and stomach cancer	Raman spectral fingerprint	AUC: 0.970	Multi-cancer classification	Require quality spectral data	[55]
14	CNN	Automated IHC HER2-stained slide	Breast cancer	IHC image system	Accuracy: 84.7%	Quick diagnosis	Limited datasets	[56]
15	RF and MLP	Microfluidic biochip	Lung cancer	Fluorescence imaging	Sensitivity: 96.7%, response: 30 min, and specificity: 100%	Quick diagnosis	Complex chip fabrication	[57]
16	YOLO-V8	Integrated microfluidic exosome chip	Exosome tumors	Fluorescence imaging	LOD: 8.65/µL	Fully automated detection	Requires optical setup	[58]
17	ResNet	Microfluidic biochip	Lung cancer	Raman spectral fingerprint	Accuracy: 97.88% and AUC: 0.95	High classification	Complex instrumentation	[59]
18	CNN	Smartphone-based microfluidic chip	Diabetes	Colorimetric response	Accuracy: 95%	Portable and low-cost	Dependent on lighting environment	[60]
19	RF	Flexible microfluidic electrochemical biochip	Diabetes	Electrochemical detection	Accuracy: 90% and LOD: 0.4 mM	High stability, wearable, and real-time monitoring	Fabrication complexity	[61]
20	SVM, SVD, and CNN	Microwave chip	Diabetes	Dielectric property variation	Linear range: 0 to 300 mg/dL	Non-invasive and no blood sampling	Environment variations	[62]

Abbreviations: CFU, colony-forming unit; LOD, limit of detection; No., number; and Ref., reference.

**Table 4:** Overview of AI-integrated biochips for neurological diagnosis and drug delivery.

Sl. No.	AI Algorithm	Platform/Biochips Type	Target Disease	Sensor Method	Performance	Advantages	Limitations	Ref.
1	ANN	Microfluidic-integrated nanoplasmonic biochip	Neurodegenerative diseases	Infrared sensor array	Accuracy: 94.66%	Label-free and multiplex detection	Complex instrumentation	[63]
2	DL	Multilayer Au nanowire	Alzheimer’s disease	Raman spectral fingerprint	Accuracy: 99.5%	Non-invasive sensing	Signal interference from biofluids	[64]
3	RF and SVM	Poly- (dimethylsiloxane)-based microfluidic plate	Alzheimer’s disease	Electrochemical sensor array	AUC: >0.94	Ultra-sensitive and multiplex biomarker detection	Fabrication complexity	[65]
4	LR, RF, and MLP	Electrophysiological microfluidic biochip	Alzheimer’s disease	Electrochemical sensor array	Accuracy: 83%	Real-time monitoring	Complex system	[66]
5	RF	Microfluidic chip	Depression and anxiety	Fluorescence-based sensor array	AUC: >0.98 and accuracy: 92%	Non-invasive sensing	Limited to specific biomarkers	[67]
6	SVM and PCA	Microfluidic multicellular co-culture array	Drug reactions for skin sensitization	Fluorescence imaging	Accuracy: 87.5% and sensitivity: 100%	Captures multicellular interactions	Smaller datasets	[68]
7	RF and SVM	Proximal tubule-on-a-chip	Tubular-interstitial fibrosis drug repurposing	Immunofluorescence	Discover 62 potentially repurposable drugs	Combines AI prediction with organ-on-chip validation	Requires animal validation	[69]
8	CNN	Biomimetic bone-on-a-chip	Osteoporosis drug testing of bone	Fluorescence imaging	Accuracy: 97.2 to 99.5% and AUC: 0.99 to 1.00	Finding side effects of drugs	Numerical AI metrics are not clear	[70]
9	SegNet and HypoNet	Alveolus-on-a-chip	High-altitude pulmonary edema drug screening	Fluorescence imaging	Accuracy: 88.9% and AUC: 00.97	Automated and high-content phenotypic drug screening	Limited physiological relevance	[71]
10	MLP	Heart-on-a-chip	Cardiac drug evaluation	Electrochemical sensor array	Accuracy: 99.62% and AUC: 00.97	Integrates a flexible sensor chip	Complex system	[72]

Abbreviations: No., number; and Ref., reference.

## Data Availability

Data are contained within the article.
